# Integrated analysis of mRNAs and long noncoding RNAs in the semen from Holstein bulls with high and low sperm motility

**DOI:** 10.1038/s41598-018-38462-x

**Published:** 2019-02-14

**Authors:** Xiuge Wang, Chunhong Yang, Fang Guo, Yaran Zhang, Zhihua Ju, Qiang Jiang, Xueming Zhao, Yong Liu, Han Zhao, Jinpeng Wang, Yan Sun, Changfa Wang, Huabin Zhu, Jinming Huang

**Affiliations:** 1 Dairy Cattle Research Center, Shandong Academy of Agricultural Sciences, Jinan, 250131 P.R. China; 2 Embryo Biotechnology and Reproduction Laboratory, Institute of Animal Sciences, Chinese Academy of Agricultural Sciences, Beijing, 100193 P.R. China; 3 College of Life Sciences, Shandong Normal University, Jinan, 250014 P.R. China

## Abstract

Sperm motility is the main index used to assess the quality of bull semen. It may also be used to evaluate the fertility potential of bulls. Protein-coding mRNA and long noncoding RNA (lncRNA) participate in the regulation of spermatogenesis. Here, we employed strand-specific RNA sequencing to profile the semen transcriptome (mRNA and lncRNA) of six paired full-sibling Holstein bulls with divergent sperm motility and to determine the functions of mRNA and lncRNA in sperm motility. Among 20,875 protein-encoding genes detected in semen, 19 were differentially expressed between the high sperm motility group (H: H1, H2, and H3) and low sperm motility group (L: L1, L2, and L3). Of the 11,561 lncRNAs identified in sperm, 2,517 were differentially expressed between the H and L groups. We found that TCONS_00041733 lncRNA targets the node gene EFNA1 (*ephrin A1*), involved in male reproductive physiology. Our study provides a global mRNA and lncRNA transcriptome of bull semen, as well as novel insights into the regulation of neighboring protein coding by lncRNAs and the influence of mRNAs on sperm motility.

## Introduction

Bull fertility is the most important factor that influences the genetic quality of the dairy herd and plays a key role in the genetic improvement of the modern cattle population. Nevertheless, over the past several decades, the dairy cow industry has neglected bull fertility in favor of milk production as its main breeding objective^1^. Semen quality is usually assessed on the basis of several characteristics, such as ejaculate volume, density, motility, and deformity rate. Among these characteristics, sperm motility is the major index applied to determine whether fresh semen can be used to produce frozen-thawed semen. Inadequate sperm motility is one of the critical reasons for male subfertility or infertility^2^. Failure in semen production due to poor sperm motility can result in significant economic loss. Usually, this is a practical problem that there is a time interval (10–12 months) from the genomic evaluation of bull to semen quality can be evaluated^3^. In particular, full-sibling Holstein bulls may have different sperm motilities despite their similar genetic backgrounds and estimated genomic breeding values. Estimated heritability for sperm motility of dairy bull is 0.43^4^. However, the molecular mechanisms underlying the difference in the sperm motilities of different bull individuals remain poorly understood.

In several genome-wide association studies, candidate genes and single nucleotide polymorphisms underlying sperm motility have been identified in bulls^5–9^. Moreover, high-throughput technologies, including transcriptomics and proteomics, have revealed a lot of differences between the spermatozoa of high and low fertility bulls^10–12^. However, there is currently little insights on which regulatory mechanisms affect the difference of bull sperm motility. Therefore, the possible reason underlying the variation in sperm motility among bulls must be investigated to improve bull fertility.

A large number of unique coding and noncoding transcripts are specifically retained in mature sperm cells and have potential regulatory roles in fertilization, early embryo development, and spermatogenetic processes^13,14^. Notably, nonprotein-coding transcripts account for a greater percentage of the transcriptomic profile than protein-coding genes. In addition to various small noncoding RNAs (e.g. miRNAs, siRNA, and piRNAs), the transcriptome can generate a large class of long noncoding RNAs (lncRNAs) that exceed 200 nucleotides in length. lncRNAs were originally considered as “junk” genomic sequences but have recently been acknowledged to regulate gene expression. However, a growing body of evidence demonstrates that up to 90% of the noncoding transcripts in the human genome have important and diverse biological roles^15^. For example, lncRNAs regulate different genes at the transcriptional, posttranscriptional, and epigenetic levels by interacting with DNA, RNA, and proteins^16–18^. The forms of lncRNAs may vary in accordance with their genomic locations and include overlapping, intergenic, intronic, sense, and antisense transcripts^19^.

Recent RNA sequencing (RNA-Seq) studies have shown that several lncRNAs may regulate testis development and spermatogenesis through currently unknown mechanisms. For example, Mrhl lncRNA affects spermatogenesis^20^, and HongrES2 regulates sperm maturation^21^. Tsx is critical for the progression of spermatocyte meiosis and is specifically expressed in pachytene spermatocytes^22^. Dmrt1 is a testis-specific functional lncRNA that participates in the transition of germ cells from mitosis to meiosis^23^. However, the functions and regulatory mechanisms of lncRNAs remain unknown^16,24^. Moreover, studies on functional lncRNAs that affect bull sperm motility have not been reported. We attempted to address this research gap in our present study. We designed this study to obtain preliminary knowledge on the molecular mechanism that controls sperm motility. We performed the high-throughput sequencing of the bull semen transcriptome to identify the functional transcripts that are involved in bull sperm motility. Our data also provide new insights into the functional effects of lncRNAs on improving sperm motility and have implications for the development of the lncRNA-based molecular breeding of dairy cows.

## Results

### Transcriptome sequencing of mRNAs and lncRNAs

**Figure 1:**
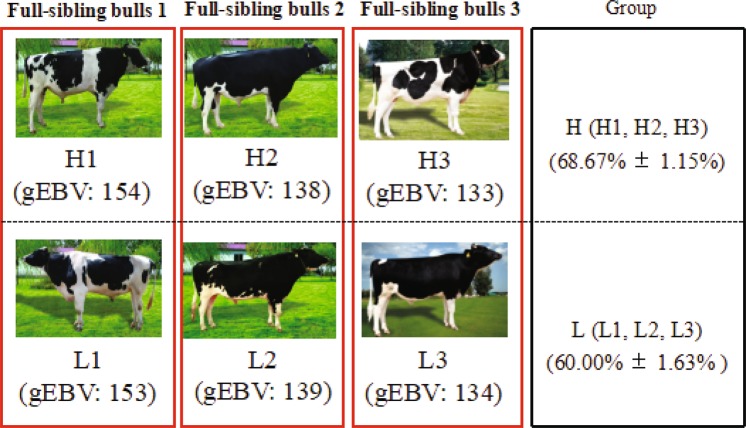
Three groups of paired full-sibling Holstein bulls. gEBV: genomic estimated breeding value. H: high sperm motility group, L: low sperm motility group. Data are presented as mean ± SE.

Three paired full-sibling Holstein bulls were selected from a bull station (Fig. 1). To construct the mRNA and lncRNA transcriptome profile of bull semen, we constructed the RNA-Seq sequencing libraries of six Holstein semen samples collected from the H and L groups. Through Illumina paired-end sequencing after strict filtering and removing low-quality raw reads, we acquired approximately 536 million clean reads that yielded 80.5 Gb high quality data. All Q20 (%) values of the read sequences in six samples exceeded 98% (Supplementary Tables S1-S2).

### **Expressed** patterns of mRNAs and lncRNAs

**Figure 2:**
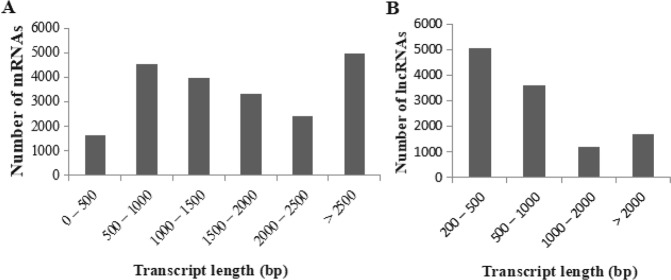
Genomic characteristics of mRNAs and lncRNAs. (**A**) Length distribution of coding mRNAs. (**B**) Length distribution of lncRNAs.

We detected 2,0875 protein-coding genes when the remaining tRNAs, miscRNAs, and other smaller RNAs were filtered out. We used CPC and CNCI and strict filtering criteria to predict the coding potential of assembled transcripts. Finally, we identified 11,561 candidate novel lncRNAs that we further categorized into four categories (Supplementary Fig. S1). Statistical analysis showed that the transcripts of the candidate lncRNAs were shorter than those of mRNAs. The length range of lncRNAs was 200–1000 bp, which was narrower than the length range of mRNAs (Fig. 2). Approximately 36.2% of the protein-coding transcripts contained more than 10 exons, whereas no more than seven exons were detected in lncRNAs.

**Figure 3:**
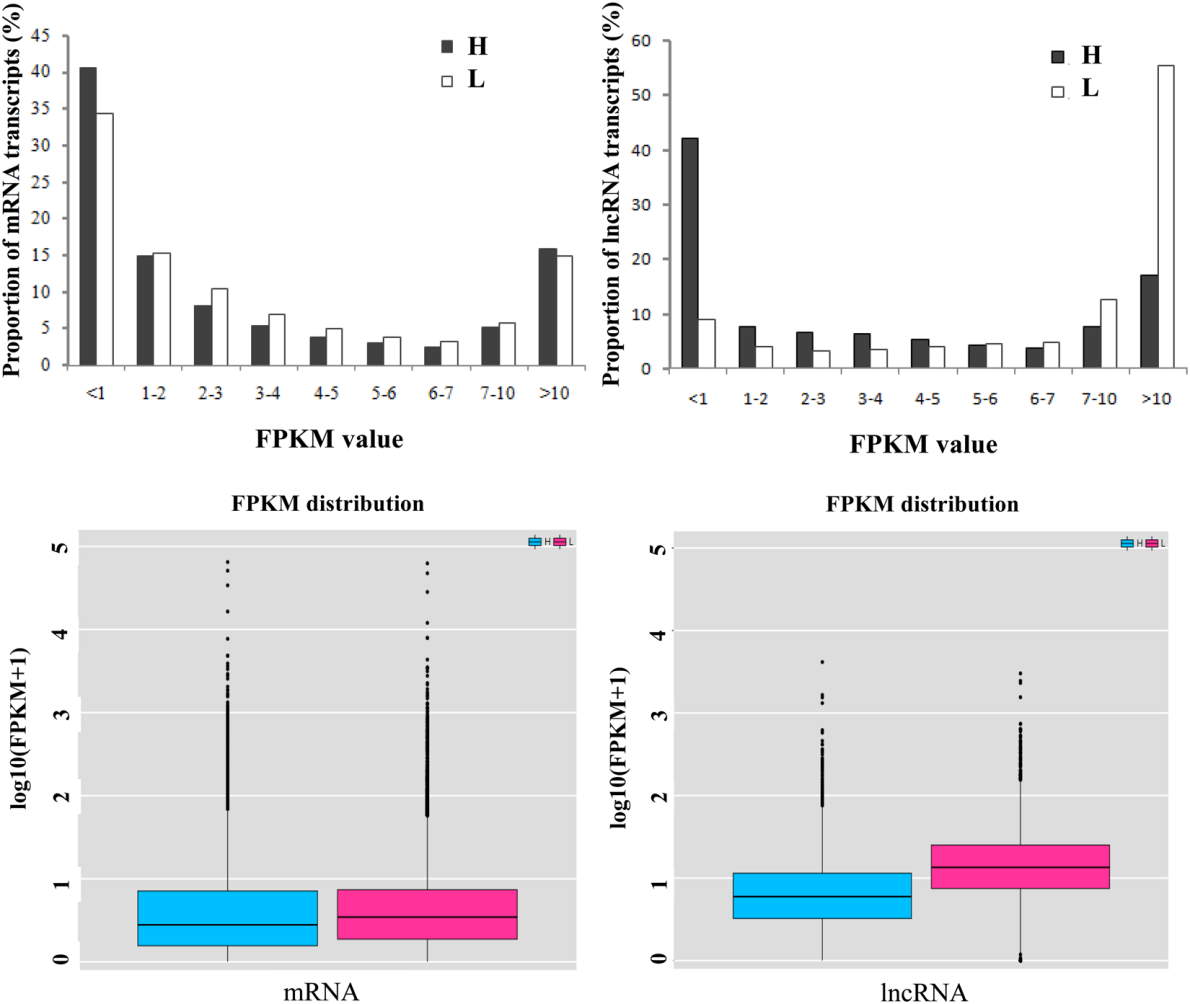
Expression levels of mRNAs and lncRNAs in the high sperm motility group (H) and low sperm motility group (L). Expression levels of mRNAs and lncRNAs in the H and L groups were compared through the FPKM method. Boxplots of FPKM distribution were calculated using all the mRNAs and lncRNAs expression data respectively. FPKM: fragments per kilobase of exon per million mapped fragments.

We estimated mRNA and lncRNA expression levels through the FPKM method by using the Cuffdiff program in Cufflinks package. We estimated the expression levels of lncRNA transcripts through FPKM. Approximately 80% of lncRNA transcripts had FPKM values of less than 2, and only approximately 10% had FPKM values of more than 10 (Fig. 3).

### Differentially expressed mRNAs and lncRNAs

Interestingly, the expression levels of lncRNA transcripts were considerably higher than those of mRNAs (Fig. 3). Among 20,875 coding genes, 19 were differentially expressed between the H and L groups, including 9 up-regulated genes and 10 down-regulated genes (FDR ≤ 0.05, |log_2_(fold change)| ≥ 1) (Table 1; Supplementary Fig. S2; Supplementary Table S3). Among 11,561 lncRNAs, 2,517 were differentially expressed between the H and L groups. These lncRNAs included 57 up-regulated genes and 2,460 down-regulated genes (FDR ≤ 0.05 and |log_2_(fold change)| ≥ 1) (Supplementary Fig. S2; Supplementary Table S4). The top 20 significantly differentially expressed lncRNAs were shown in Table 2. To validate the results from the RNA-Seq data, we verified the expression levels of ten differentially expressed genes that were randomly selected from sequencing data, including five mRNAs and five lncRNAs, through qRT-PCR analysis. Our analysis indicated that the gene expression patterns validated through qRT-PCR are consistent with the results of RNA-Seq high-throughput sequencing (Supplementary Fig. S3; Supplementary Tables S5–S6).

**Table 1:** The 19 significantly differentially expressed genes. Note: inf means positive infinity; −inf means negative infinity.

gene	Ensembl Gene ID	locus	H(FPKM)	L(FPKM)	log2(fold_change)	FDR_value
NKX1-2	ENSBTAG00000020918	chr26:44416759-44420132	0.00	5.46	−inf	0.01
AQP2	ENSBTAG00000008374	chr5:30094769-30103142	46.69	5.40	3.11	0.02
SUBH2BV	ENSBTAG00000012775	chr20:8803855-8804333	369.16	104.00	1.83	0.02
SHD	ENSBTAG00000039696	chr7:21002692-21010652	87.36	261.12	−1.58	0.02
EFNA1	ENSBTAG00000020244	chr3:15521493-15528212	107.24	17.86	2.59	0.02
SESN3	ENSBTAG00000004034	chr15:15502202-15530863	28.12	9.61	1.55	0.02
RPS27	ENSBTAG00000013866	chr3:16505731-16507247	1323.87	512.66	1.37	0.02
U1	ENSBTAG00000048270	chr18:14877691-14877845	329.93	0.00	inf	0.03
ENSBTAG00000038351	ENSBTAG00000038351	chr5:441592-442266	0.00	158.13	−inf	0.03
ENSBTAG00000046783	ENSBTAG00000046783	chr2:94202054-94202354	21.49	0.00	inf	0.03
PCDH8	ENSBTAG00000018179	chr12:10938750-10943209	2.09	11.90	−2.51	0.04
RPL30	ENSBTAG00000040051	chr14:68467562-68470952	474.20	226.19	1.07	0.04
RBMX	ENSBTAG00000047652	chr15:46287139-46288701	4.61	18.43	−2.00	0.04
PRDM13	ENSBTAG00000006095	chr9:50873668-50881408	1.84	11.47	−2.64	0.04
FZD1	ENSBTAG00000002107	chr4:8490675-8494829	1.41	6.80	−2.27	0.05
GTSF1L	ENSBTAG00000011233	chr13:72984199-72985097	318.49	142.18	1.16	0.05
MLPH	ENSBTAG00000000634	chr3:117584072-117637812	1.62	7.29	−2.17	0.05
SLAIN2	ENSBTAG00000021963	chr6:68695297-68741224	16.18	40.23	−1.31	0.05
RBM46	ENSBTAG00000001696	chr17:2523017-2567620	4.14	16.58	−2.00	0.05

**Table 2:** The top 20 significantly differentially expressed lncRNAs. Note: inf means positive infinity; −inf means negative infinity.

lncRNAs	locus	H(FPKM)	L(FPKM)	log2(fold_change)	FDR_value
TCONS_00000538	chr1:75937027-75937410	0.00	80.73	−inf	0.01
TCONS_00003123	chr10:55232037-55232318	0.00	153.91	−inf	0.01
TCONS_00005494	chr11:62030429-62030804	0.00	45.96	−inf	0.01
TCONS_00007217	chr11:97395420-97396011	0.00	7.90	−inf	0.01
TCONS_00008747	chr12:87789106-87792905	20.13	0.00	inf	0.01
TCONS_00009226	chr13:46843141-46843647	0.00	21.71	−inf	0.01
TCONS_00011237	chr14:6662538-6663034	0.00	18.56	−inf	0.01
TCONS_00012048	chr14:6722290-6722813	0.00	20.09	−inf	0.01
TCONS_00012181	chr14:23115327-23115826	0.00	13.76	−inf	0.01
TCONS_00012775	chr15:19466827-19467304	0.00	19.09	−inf	0.01
TCONS_00013706	chr15:85036155-85036516	0.00	14.22	−inf	0.01
TCONS_00014744	chr16:2115585-2116115	15.22	0.00	inf	0.01
TCONS_00014816	chr16:7166876-7167359	0.00	16.80	−inf	0.01
TCONS_00015123	chr16:43687434-43687736	0.00	32.20	−inf	0.01
TCONS_00019053	chr18:41851514-41851878	0.00	35.39	−inf	0.01
TCONS_00020130	chr18:16232385-16233355	10.72	0.00	inf	0.01
TCONS_00021031	chr18:64429371-64429857	24.37	0.00	inf	0.01
TCONS_00021657	chr19:34327860-34328423	0.00	16.26	−inf	0.01
TCONS_00024090	chr2:18693367-18693630	0.00	31.45	−inf	0.01
TCONS_00025506	chr2:24451811-24452412	0.00	15.76	−inf	0.01

### Chromosomal mapping of mRNAs and lncRNAs

**Figure 4:**
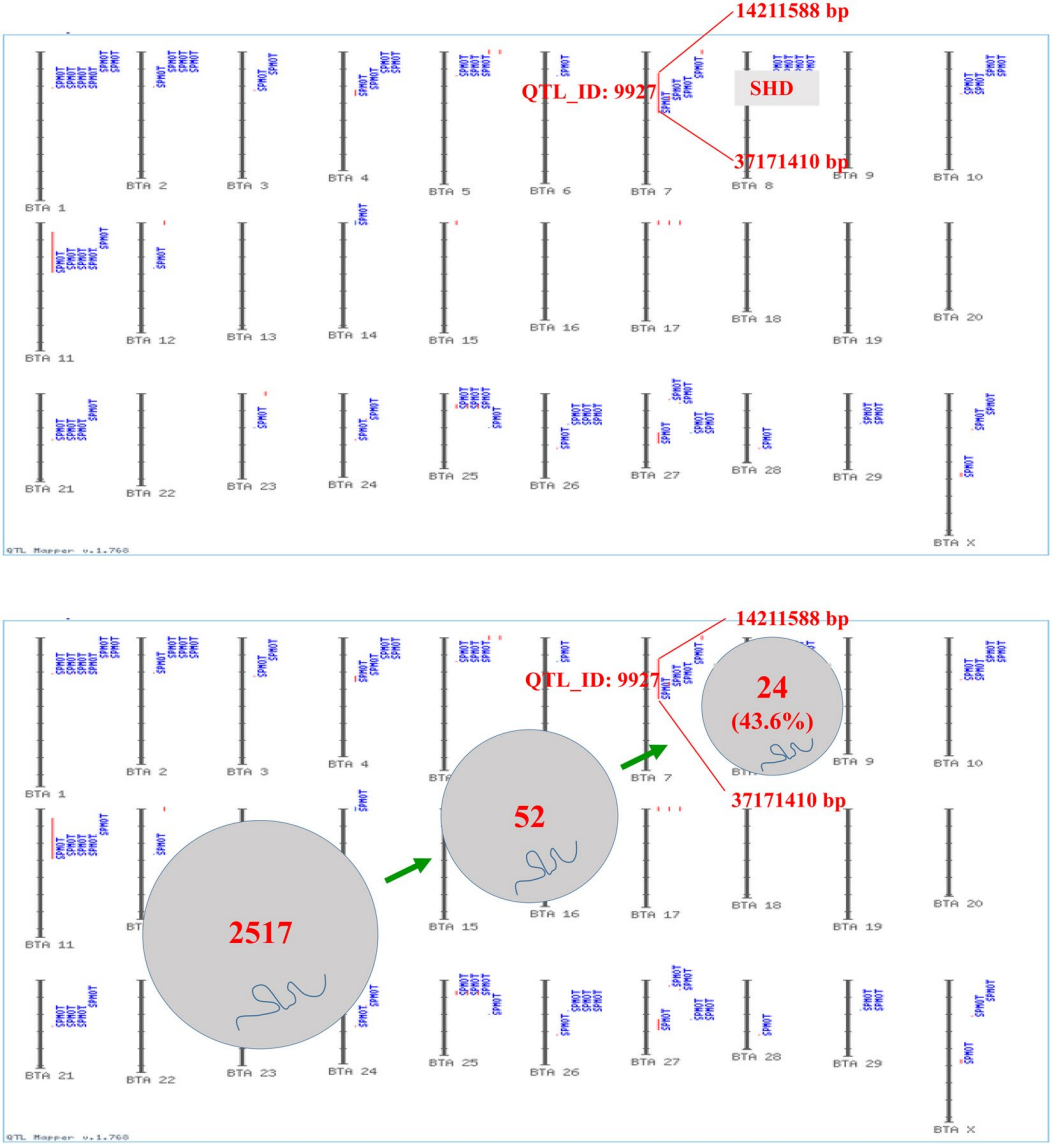
Mapping of differentially expressed mRNAs and lncRNAs on QTLs related to sperm motility.

First, we analyzed the chromosomal distribution of 19 differentially expressed coding genes and 2,517 differentially expressed lncRNAs. Statistical results showed that differentially expressed coding genes were mostly located on chromosomes 3, 5 and 15 and differentially expressed lncRNAs were mostly located on chromosomes 1, 2 and 6 (Supplementary Fig. S4). Then, we mapped differentially expressed mRNAs and lncRNAs to cattle quantitative trait loci (QTLs) (http://www.animalgenome.org/cgi-bin/QTLdb/BT/index). We found that differentially expressed coding gene SHD could be mapped onto a QTL (QTL_ID: 9927) related to sperm motility on chromosomes 7. Moreover, 52 out of 2,517 lncRNAs were located on cattle QTLs related to sperm motility, among which 46.2% (24/52) lncRNAs were mapped to the QTL (QTL_ID: 9927) on chromosome 7 (Fig. 4, Supplementary Table S7).

## Discussion

With the development of accurate and cost-effective high-throughput genomic analysis technology, many critical genes responsible for species characteristics and regulatory pathways that underlie biological processes have been unraveled^25^. Herein, we constructed the expression profiles of mRNAs and lncRNAs in the H and L groups and comprehensively analyzed and identified the candidate key genes and functional pathways that are related to sperm motility. The basic profiles of the genes revealed notable differences between the H and L groups.

As a result, we detected 19 differentially expressed mRNAs and 2,517 differentially expressed lncRNAs between the H and L groups. We also identified 5 differentially expressed genes including RBMX, AQP2, EFNA1, MLPH and RPL30, that are involved in the “extracellular exosome” GO term. In boars, seminal plasma exosomes prolong the effective motility time of sperm by directly binding to the membrane of the sperm head; this action could improve sperm plasma membrane integrity^26^. Parts of differentially expressed genes are also reported being involved in spermatogenesis, sperm motility, and fertilization. For example, previous studies have revealed that RBMX which acted as the role of potential splicing factors could regulate spermatogenesis^27^; AQP2 is expressed in seminiferous tubules of the testis, localized to Leydig cells and elongated and round spermatids, which may be pivotal for establishment of male fertility^28,29^. We also found that differentially expressed gene SHD and 52 lncRNAs are located on cattle QTLs related to sperm motility. Moreover, approximately 46.2% (24/52) of lncRNAs are mapped to a QTL (QTL_ID: 9927) on chromosome 7. The colocation of these lncRNAs indicates that these differentially expressed genes may influence sperm motility through their complex interactions.

Interestingly, our finding showed that the number of differentially expressed lncRNAs is much greater than that of mRNAs in mature sperm of bull. Increasing studies have shown that specific lncRNAs expressed in mature sperm and testis may be related to sperm motility. Recent research revealed that there was an association between lncRNA expression and sperm motility by analyzing the lncRNA expression profiles in human mature sperm^30^. Previous study has also identified several spermatogenic lncRNA that are differentially expressed in testis of sheep which were associated with sperm motility^31^. Analogously, compared with mRNAs, the number of differentially expressed lncRNAs is much greater in sperm by exposure to cadmium^32^. Therefore, the above data suggest that lncRNA may be as a promising candidate marker for assessing bull sperm motility. Continued studies should be carried out to explore other utilities of bull sperm lncRNAs, such as fertility prediction and assisted reproductive technologies^33^.

A major function of lncRNAs is the regulation of the expression of neighboring protein-coding genes via transcriptional coactivation/repression^34–36^. Therefore, we searched and identified differentially expressed mRNAs that are located within the 100 kb upstream and downstream regions of differentially expressed lncRNAs as cis target genes. We found that the identified differentially expressed lncRNA TCONS_00041733 may regulate differentially expressed coding gene EFNA1 (*ephrin A1*). EFNA1 is a cell surface protein and is involved in vascular development^37^, inflammatory response and cell proliferation and angiogenesis^38^ as well as sperm morphology and membrane functionality^39^. EFNA1 was higher expressed in sperm, seminal veiscle fluid and seminal plasma of bulls^39–41^. Higher levels of EFNA1 may possible result in an attack of immune cells over the sperm and therefore damage the cell, but the precise role of EFNA1 in male reproductive physiology needs to be confirmed^39^. Taken together, the EFNA1 gene can be as a candidate gene affecting the sperm motility based on our data. Therefore, further study should be carried out in the next experiment.

In this study, we aimed to provide a global expression profile of mRNAs and lncRNAs in bull semen. However, as a limitation in the data analysis, specially these expressed genes with the low value of FPKM were not excluded in the process of gene differential expression analysis, which may cause false positive results. Therefore, these lowly expressed mRNAs and lncRNAs should be carefully verified in future experiments. The other limitation was that only a small number of lncRNAs and targets were highlighted due to the limited available annotation and unknown functions of gene.

## Conclusion

We used the RNA-Seq method to reveal for the first time that the expression patterns of lncRNAs and mRNAs in semen from bulls with high sperm motility are different from those of lncRNAs and mRNAs in semen from bulls with low sperm motility. We identified differentially expressed mRNAs and lncRNAs that may potentially influence bull sperm motility. Additionally, aberrantly expressed lncRNAs could regulate the expression of protein-coding genes and pathways that may contribute to bull sperm motility. Although our findings provide a valuable foundation for further research on the role of lncRNAs in sperm motility, further studies are also required to fully elucidate the detailed molecular mechanisms that underlie the function and contribution of lncRNAs to the characteristics of sperm quality.

## Materials and Methods

### Ethics statement

All experiments involving animals were conducted in accordance with the Regulations for the Administration of Affairs Concerning Experimental Animals published by the Ministry of Science and Technology of China in 2004 (http://www.most.gov.cn/fggw/zfwj/zfwj2006/200609/t20060930_54389.htm). Our studies were approved by the Animal Care and Use Committee of the Dairy Cattle Research Center, Shandong Academy of Agricultural Sciences (Shandong, China).

### Animals and sample collection

Three paired full-sibling Holstein bulls (4.3–5.8 years old) produced through embryo transfer were selected from a bull station (Jinan City, Shandong Province, China). Each pair of bulls had approximately equal genomic estimated breeding values (GISU) (http://idele.fr/fileadmin/medias/Documents/INTRO_NO_133_va.pdf), which were calculated by the Animal Genetics Department of INRA (National Institute for Agronomic Research) and genomic evluation data were provided by Genes Diffusion (Douai Cedex, France). However, their sperm motility phenotypes, as determined with a sperm analysis system (AndroVision, Minitube, Germany), were different (p < 0.05, Fig. 1). According to the average sperm motility from the production record from 2011 to 2015, three bulls were allocated to the high sperm motility group (H: H1, H2, and H3), and the three remaining bulls were allocated to the low sperm motility group (L: L1, L2, and L3). Prior to the sampling of semen for RNA-seq, the collected semen were assessed three times in a row to confirm the grouping. The sperm motility of the H and L groups were 68.67% ± 1.15% and 60.00% ± 1.63% (mean ± SE, Fig. 1), respectively. Fresh semen were collected individually from each bull and immediately frozen in liquid nitrogen for storage until RNA extraction.

### RNA extraction, library construction, and sequencing

Total RNA was extracted from each fresh semen sample by using Animal Tissue RNA Purification Kit TRK-1002 (LC Sciences, Houston, Texas) in accordance with the manufacturer’s instructions. RNA quality was evaluated using Agilent 2100 Bioanalyzer (Agilent Technologies, Santa Clara, CA, USA) and Nano Drop ND-2000 Spectrophotometer (Nano-Drop, Wilmington, DE, USA). The value of RNA integrity number exceeded 7.0, and samples with OD_260/280_ values of more than 1.8 were used for the following steps.

Total RNA was randomly fragmented into small pieces and purified into fragments with lengths of 200–600 bp. Cleaved RNA fragments were subsequently reverse-transcribed to create the final RNA-seq library, which was then sequenced by using the Illumina HiSeq^TM^ 4000 platform with paired-end sequencing (2 × 150 bp read length) following the vendor’s recommended protocol. Raw sequencing data in the fastq format were submitted to the NCBI SRA database (http://www.ncbi.nlm.nih.gov/Traces/sra), with accession number SRP158901.

### Transcriptome assembly

Raw image data from sequencing machines were transformed through base calling into sequence reads, which were designated as raw reads. The obtained sequence raw reads in FASTAQ file format were first checked by FastQC software. Then, the raw reads were filtered through following process: (1) Reads with adapter contamination were first eliminated. (2) Reads with unknown bases larger than 5% were discarded. (3) Reads with more than 20% of bases with a quality score of Q ≤ 10 were removed. After the above steps, clean reads were produced from raw reads for subsequent analysis.

All clean reads were mapped to the cow reference genome database (ftp://ftp.ensembl.org/pub/release-86/fasta/bos_taurus/dna/) by Tophat2 software (v2.0.9) with a mismatch tolerance of three nts^42^. To construct the transcriptome, the mapped reads of each sample were assembled using Cufflinks (v2.1.1) package^43^. Then, Cuffmerge program was used to merge all transcriptomes from six samples to reconstruct a comprehensive transcriptome.

### Identification of lncRNAs

Assembled transcripts were annotated using the Cuffcompare program in the Cufflinks package^44^. Transcripts that overlap with known mRNAs were first excluded. Then, the coding potential of remaining transcripts with length ≥200 bp, mapped read coverage ≥3, and exon number ≥1 was calculated by Coding Potential Calculator (CPC) and Coding-Non-Coding-Index (CNCI). CPC assesses the protein-coding potential of transcripts on the basis of six biologically meaningful sequence features by using a classifier based on the support vector machine^45^. CNCI effectively classifies protein-coding or noncoding transcripts by profiling adjoining nucleotide triplets independent of known annotations^46^. If the predicted results were CPC score <−1 and CNCI score <0, the transcript was considered as a candidate lncRNA.

### Differential expression analysis

To eliminate the influence of different transcript lengths and sequencing levels, the expression levels of mRNAs and lncRNAs in each sample were calculated as fragments per kilobase of exon per million mapped fragments (FPKM) by using the Cuffdiff program in Cufflinks package^44^. The formula used was FPKM = (106 C)/(NL/103), where C is the number of fragments aligned with unigene exons, N is the number of total fragments aligned with all unigenes, and L is the length of exons in base pairs. Here, the levels of differential mRNA and lncRNA expression were analyzed through Cuffdiff on the basis of negative binomial distribution. The statistical significance of the differences in expression levels between two groups of semen was analyzed using Student’s *t*-test.

### Target gene prediction

Increasing evidence has indicated that lncRNAs may affect the expression of neighboring coding genes by playing a cis regulating role^47,48^. To explore the potential function of lncRNAs, differentially expressed lncRNAs were selected for target gene prediction. To reduce the rate of false positives, we did not analyze the trans role of lncRNAs. We only selected differentially expressed mRNAs as potential target genes of lncRNAs. Moreover, we searched mRNAs within the 100 kb upstream and downstream regions of differentially expressed lncRNAs as the cis target genes.

### Real-time PCR validation

Real-time PCR (qRT-PCR) was performed on an LightCycler 480II Real-Time PCR System using SYBR® Green PCR Master Mix (TaKaRa, Dalian, China) with specific primers. Gene expression levels were normalized to beta-actin gene expression through the 2^−ΔΔCt^ method. Each reaction was performed with a 25 μL reaction mixture that contained 12.5 μL of 2 × SYBR Green Real-time PCR Master Mix, 1 μL of each primer, 2 μL of cDNA, and 8.5 μL of H_2_O. PCR cycling conditions were as follows: an initial single cycle at 95 °C for 3 min followed by 40 cycles at 95 °C for 15 s, 57 °C for 15 s, and 72 °C for 20 s. Three independent biological replicates were used for each reaction.

## Supplementary information

Supplementary Fig. S1-S4

Table S1

Table S2

Table S3

Table S4

Table S5

Table S6

Table S7
