# Supplementary material for: Integrated analysis of mRNAs and long noncoding RNAs in the semen from Holstein bulls with high and low sperm motility
Source: Sci Rep. 2019 Feb 14;9:2092. doi: 10.1038/s41598-018-38462-x (PMC6376035; doi:10.1038/s41598-018-38462-x)

Supplementary Information

Integrated analysis of mRNAs and long noncoding RNAs in the semen from Holstein bulls with high and low sperm motility

Xiuge Wang[1,#], Chunhong Yang[1,#], Fang Guo[1], Yaran Zhang[1], Zhihua Ju[1], Qiang Jiang[1], Xueming Zhao[2], Yong Liu[1], Han Zhao[3], Jinpeng Wang[1], Yan Sun[1], Changfa Wang[1], Huabin Zhu[2], Jinming Huang[1,3,*]

Running head: lncRNA affecting bull sperm motility

[1] Dairy Cattle Research Center, Shandong Academy of Agricultural Sciences, No. 159 Gongye North Road, Jinan, 25131, P.R. China.

[2] Embryo Biotechnology and Reproduction Laboratory, Institute of Animal Sciences, Chinese Academy of Agricultural Sciences, Beijing, 100193, P.R. China.

[3] College of Life Sciences, Shandong Normal University, Jinan, 250014, P.R. China.

[#] These authors equally contributed to the study.

* Correspondence and requests for materials should be addressed to J.M. Huang. (email: huangjinm@sina.com)

Figure S1. Categories and density distribution of novel assembled lncRNAs. (A) Distribution of “u”, “o”, “x”, and “j” categories. (B) Density distribution of the “j”, “o”, “u”, and “x” lncRNA categories on the cow chromosome.

A

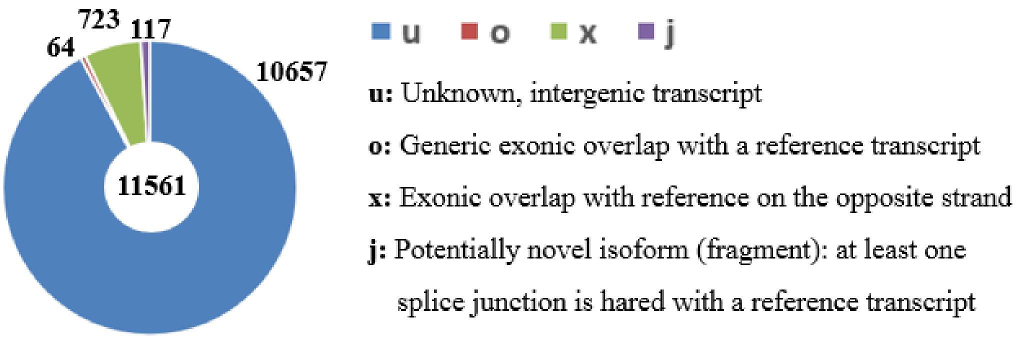

B

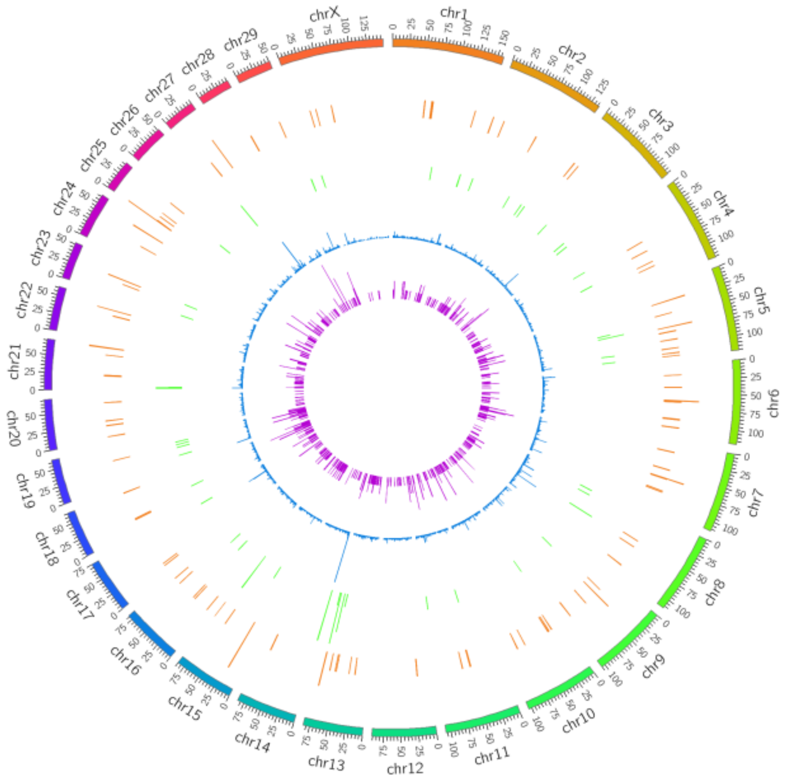

Figure S2. Expression levels of mRNAs and lncRNAs in the high sperm motility group (H) and low sperm motility group (L). Analysis of differentially expressed mRNAs and lncRNAs.

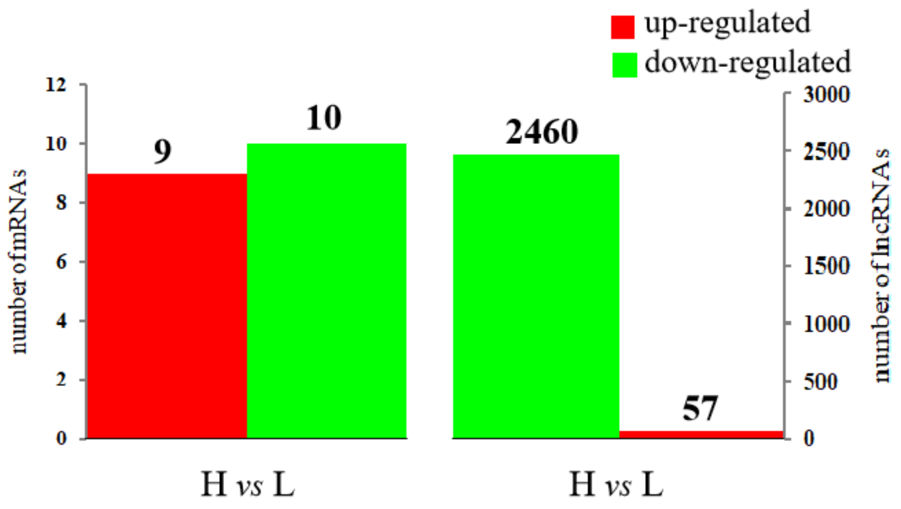

Figure S3. Verification of RNA-Seq mRNA expression results through qRT-PCR. Five mRNA genes expressed in semen were selected for validation. Data are presented as the difference in relative expression between the H and L groups. The expression of each gene was normalized to that of β-actin. Bars represent the mean (± SD) of three experiments.

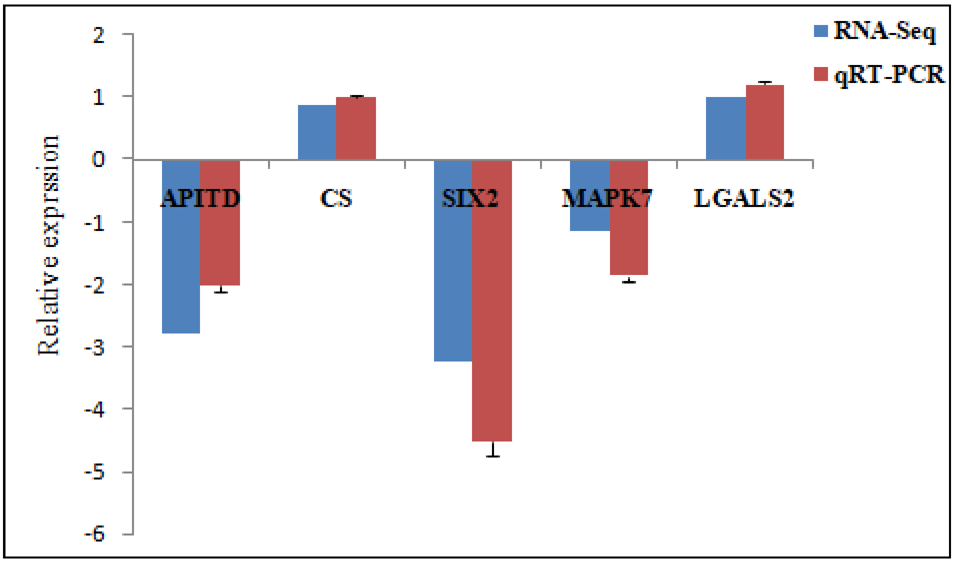

Figure S4. Chromosomal distribution of differentially expressed coding genes and lncRNAs. (A) Chromosomal distribution of 281 differentially expressed coding genes. (B) Chromosomal distribution of 2602 differentially expressed lncRNAs.

A

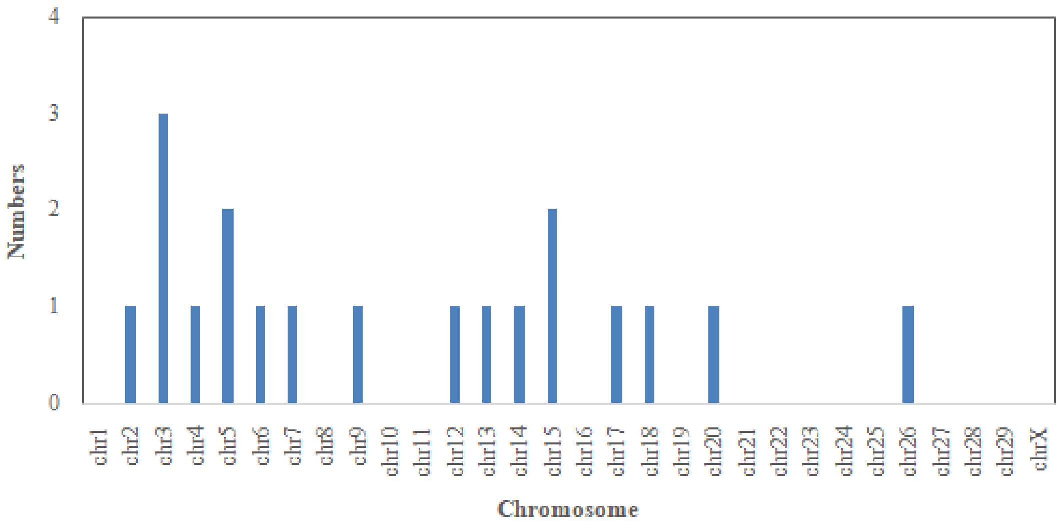

B

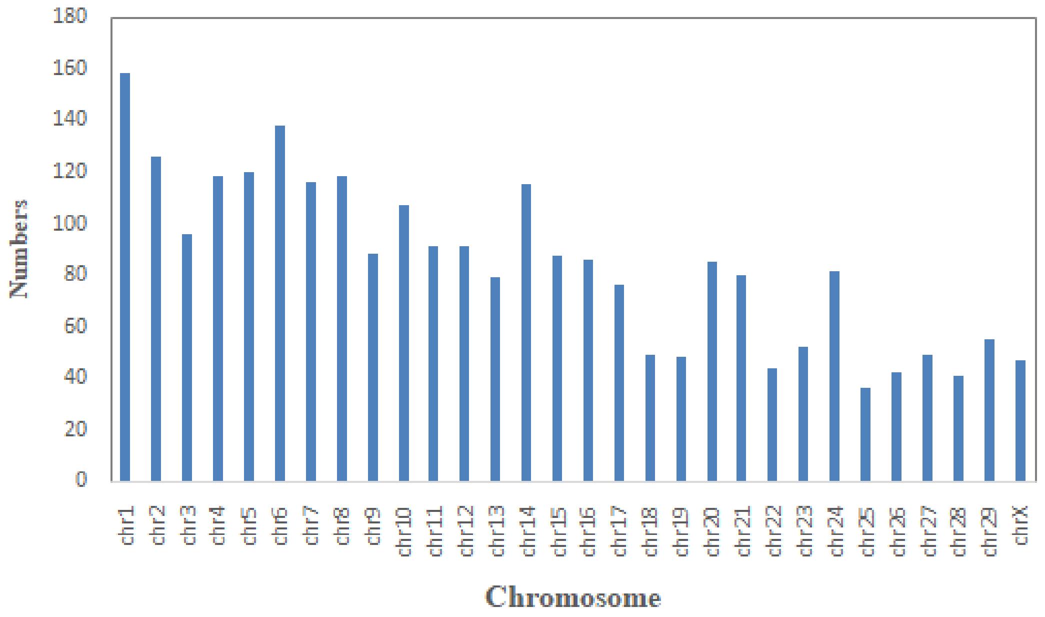

Supplement: Supplementary file 1 — Supplementary Fig. S1-S4 [file 41598_2018_38462_MOESM1_ESM.pdf]
